# Reasons and predictors for antiretroviral therapy change among HIV-infected adults at South West Ethiopia

**DOI:** 10.1186/s13104-018-3470-y

**Published:** 2018-06-05

**Authors:** Endalkachew Mekonnen, Abdulhalik Workicho, Nezif Hussein, Teka Feyera

**Affiliations:** 1 Department of Medicine, College of Medicine and Health Science, Jigjiga University, Jigjiga, Ethiopia; 2 Department of Epidemiology, College of Health and Medical Sciences, Jimma University, Jimma, Ethiopia; 3 School of Pharmacy, College of Health and Medical Sciences, Jimma University, Jimma, Ethiopia; 4 Department of Veterinary Clinical Studies, College of Veterinary Medicine, Jigjiga University, Jigjiga, Ethiopia

**Keywords:** Regimen change, Risk factors, Initial highly active antiretroviral therapy, Ethiopia

## Abstract

**Objective:**

This retrospective cohort study is aimed to assess reasons and predictors of regimen change from initial highly active antiretroviral therapy among 1533 Human Immunodeficiency virus-infected adult patients at the Jimma University Tertiary Hospital.

**Results:**

One in two (47.7%) adults changed their antiretroviral therapy regimen. Patients who were above the primary level of education [Hazard ratio (HR) 1.241 (95% CI 1.070–1.440)] and with human immunodeficiency virus/tuberculosis co-infection [HR 1.405 (95% CI 1.156–1.708)] had the higher risk of regimen change than their comparator. Individuals on Efavirenz [HR 0.675 (95% CI 0.553–0.825)] and non-stavudine [HR 0.494 (95% CI 0.406–0.601)] based regimens had lower risk of regimen change.

## Introduction

Globally, the number of people living with human immunodeficiency virus (HIV) reached 38.8 million in 2015 [1]. In Ethiopia, there were 786,040 HIV-infected people, 39,140 new HIV infections and 28,650 human immunodeficiency virus/acquired immunodeficiency syndrome (HIV/AIDS) related deaths in 2015 [1].

Highly active antiretroviral therapy (HAART) has led to a major reduction of HIV related morbidity and mortality [2–4]. These optimum clinical and public health achievements of antiretroviral therapy (ART) require consistent long-term adherence [5]. Currently, ART regimen changes become a big challenge and cause diminishing the clinical and immunological benefit of treatment [6, 7], failing virological suppression, increase drug resistance [8] and increase mortality and morbidity due to HIV/AIDS [8, 9].

In Africa, the magnitude of regimen change in 2009–2012 was between 13.7 and 57.4% [10, 11] and in Ethiopia, it was between 9.8 and 31.4% in 2012 and 2014 [12]. Beyond access to HAART, the long-term successes of treatment programs in resource-limited settings depend on patient retention on therapy.

The reasons and risk factors for HAART regimen changes have been assessed by studies from resource-rich and resource-limited settings [13–17]. Similarly, in Ethiopia, some studies determined factors contributed to ART regimen change [12, 18]. However, the previous studies that assessed either the prevalence [12, 18] or factors [12, 18–22] for regimen change were all from the northern part of the country. In this study, we assessed reasons and risk factors for the initial HAART regimen changed among patients who started HAART as part of routine clinical care at Jimma University Tertiary Hospital [JUTH].

## Main text

### Methods

#### Study design, settings, and participants

A retrospective data from 1533 treatment-naïve adult patients who first received HAART from 2006 to June 2014 at ART clinic of JUTH, Southwest Ethiopia, was analyzed. The treatment protocol for Ethiopia is implemented using World Health Organization (WHO) ART treatment guideline for HIV infection in adults and adolescents [23] and national guidelines for HIV prevention, care, and treatment: Federal Democratic Republic of Ethiopia [24]. According to the current treatment guidelines, HIV-infected adults are eligible to start ART if their Cluster of differentiation 4 cell (CD4cell) count is ≤ 500 cells/mm^3^ irrespective of the CD4 cell count and WHO clinical stage. Breastfeeding women, pregnant women, and serodiscordant couples can start ART irrespective of WHO clinical stages and CD4 cell count.

#### Data collection techniques and data quality control

A standard checklist containing study variables were developed from the patient registry card which was developed by the Ethiopian Federal Ministry of Health (FMOH). During data collection, the most recent laboratory results before starting ART were generally used as baseline values. Pregnant women initiated ART for prevention of mother to child transmission (PMCT) and transfer-in patients were excluded from the study. Death, transferred out and unrecorded outcome variables would be excluded from complete case analysis.

#### Study variables and measurements

Survival time was measured from the start of ART and ended at the time of regimen changed or when patients were censored. Regimen change refers to both medication modification (i.e. Patients who had modified one or two ART from the three ART regimens) and discontinuation (i.e. Patients who had stopped all regimens at once). Lost to follow up (LTFU) (i.e. Patients who had missed at least three appointments, but had not yet been classified as dead or transferred), and defaulters (i.e. Patients who had missed less than three clinical appointments, but had not yet been classified as dead or transferred) were censored.

The independent variables include: age, sex, marital status, past opportunistic infection, tuberculosis (Tb) status, baseline functional status, educational status, initial CD4 count, and WHO clinical stages. Educational status was categorized into below (1–8 grade) and above (> 9 grade) primary level education. Functional status was classified into the following categories: working (i.e. the ability to perform usual work in and out of the house), ambulatory (i.e. the ability to perform activities of daily living), and bedridden (i.e. not able to perform activities of daily living).

### Statistical analyses

The collected data were cleaned, categorized, coded, entered and analyzed by using statistical package for social sciences (SPSS) version 16. The probability of HAART regimen changes was estimated using Kaplan–Meier survival analysis. We applied Cox-proportional hazards regression to identify risk factors associated with outcome variable (time of regimen change). Variables found to be associated with the outcome in the univariate analysis assuming a significance threshold of 20% were included in the multivariate analysis. The multivariate analysis results showed a significant effect on the outcome considering the significance thresholds of 5% were described.

## Ethical consideration

Ethical clearance was obtained from Ethical clearance board of Jimma University and data access permission was obtained from the medical director of JUTH. We simply extracted anonymized data from the patient’s medical registry and no participant was involved in the study.

## Results

### Characteristics of study participants

A total of 1533 naive patients who started ART with at least one follow-up visit were included for complete case analysis, of which 963 (62.8%) were females. Socio-demographics, clinical and laboratory characteristics of the patients are shown in Table 1.

**Table 1:** Baseline socio-demographic and clinical characteristics of patients on ART at JUTH, Ethiopia 2015

Baseline characteristics	N (%)
Age (years), [interquartile range (IQR)]	30 (26–38)
Sex	
Female	963 (62.8)
Male	570 (37.2)
Marital status	
In relationship	733 (47.8)
Not in relationship	798 (52.0)
Missing values	2
Educational status	
< primary level education	835 (54.5)
≥ primary level education	698 (45.5)
Functional status	
Working	1058 (69.0)
Ambulatory	394 (25.7)
Bedridden	63 (4.1)
Missing values	18 (1.2)
Initial CD4	
Median (IQR) in cells/mm^3^	144 (IQR 79–210)
≤ 200	1102 (71.8)
> 200	429 (28)
Missing values	2
WHO clinical stages	
Stage I	290 (18.9)
Stage II	436 (28.4)
Stage III	648 (42.3)
Stage IV	159 (10.4)
Past opportunistic infections	
Yes	1183 (77.2)
No	342 (22.3)
Missing values	8
TB status	
Negative	1207 (78.7)
Positive	281 (18.3)
Missing values	45 (2.9)
NNRTIs	
NVP-based	1056 (68.9)
EFV-based	477 (31.1)
NRTIs	
D4T-based	942 (61.4)
TDF-based	338 (22.0)
ZDV-based	253 (16.5)

WHO clinical stage I indicates asymptomatic and persistent generalized lymphadenopathy; WHO clinical Stage 3 was defined if one of the following is present: weight loss of > 10% body weight, chronic diarrhea for > 1 month, fever for > 1 month, oral candidiasis, or pulmonary Tb within the previous year, or severe bacterial infections; WHO clinical Stage 4 was defined if one of the following is present in an HIV diagnosed patient: HIV wasting syndrome, (*Pneumocystcarini pneumonia*)PCP, toxoplasmosis of the brain, cryptosporidiosis with diarrhea for > 1 month, cytomegalovirus disease, herpes simplex virus infection, progressive multifocal leukoencephalopathy, candidiasis, extra-pulmonary Tb, lymphoma, kaposi’s sarcoma

*NNRTIs* non-nucleoside reverse transcriptase inhibitors, *NRTIs* nucleoside reverse transcriptase inhibitors, *NVP* Nevirapine, *EFV* Efavirenz, *D4T* Stavudine, *TDF* Tenofovir, *ZDV* Zidovudine

### Prevalence and trends of ART regimen change

Patients were followed for a total of 4546.85 PY with a median follow up of 54.20 (95% CI 51.012–57.388) months. In total, 731 (47.7%) had regimen changed corresponding to an overall incidence rate of 16.08 (95% CI 14.94–17.27) per 100 PY. The number of defaulters and LTFU adult patients were 239 (15.60%) and 107 (6.98%), respectively. Of the 731 adults who had regimen changed, 640 (87.55%) were modifications and 91 (12.45%) were discontinuations. The median time from ART initiation to regimen change was 24.37 (95% CI 21.463–27.204) months.

Kaplan–Meier estimates of the overall probabilities of regimen change at 3-month and 1-year for the cohort were 8.1% (7.4–8.8%) and 16.6% (15.6–17.6%), respectively. The next 2- and 3-year probabilities of regimen changes were 24.2% (95% CI 23.1–25.3%) and 31.2% (95% CI 30–32.4%), respectively.

Drug toxicities were the predominant reasons [n = 431 (58.96%)] for ART regimens changed. Among documented toxicities: 82 (61.19%) were fat changes, 30 (22.38%) were peripheral neuropathies, 14 (10.45%) were anemic, 5 (3.73%) were central nervous system (CNS) toxicities and one hepatotoxicity.

A new TB treatment [n = 120 (16.42%)], planning pregnancy or being pregnant [n = 29 (3.96%)] and treatment failure [n = 24 (3.28%)] were the other reasons for regimens changed. Others (n = 127) had no documented reason for regimen changed.

### Predictors for ART regimen change

The results from the multivariable Cox-proportional hazards regression analysis found patients who were above the primary level of education [HR 1.241 (1.070–1.440)] and with HIV/TB co-infection [HR 1.405 (1.156–1.708)] had a higher risk of regimen change than their comparator as shown in Table 2. Patients on EFV-based regimens [HR 0.675 (95% CI 0.553–0.825)] and non-D4T based regimens [HR 0.494 (0.406–0.601)] had the lower hazard of change than NVP and D4T based regimens, respectively.

**Table 2:** Hazard ratios (95% CI) for ART regimens change among HIV infected adult patients at JUTH, Ethiopia, 2015

Variables	Unadjusted HR (95% CI)	Adjusted HR (95% CI)
Educational status		
Below primary level education	1	1
Above primary level education	1.209 (1.046–1.398)	1.241 (1.070–1.440)
TB status		
Negative	1	1
Positive	1.356 (1.126–1.633)	1.405 (1.156–1.708)
Functional status		
Working	1	1
Ambulatory	1.196 (1.017–1.408)	1.169 (0.987–1.385)
Bedridden	1.428 (1.020–1.998)	1.369 (0.972–1.929)
Initial CD4		
≤ 200	1	1
> 200	0.850 (0.719–1.005)	0.923 (0.777–1.097)
NNRTIs-based		
NVP based	1	1
EFV based	0.736 (0.605–0.895)	0.675 (0.553–0.825)
NRTIs-based		
D4T based	1	1
Non-D4T based	0.507 (0.418–0.615)	0.494 (0.406–0.601)

## Discussion

Regimen change affects the success of the treatments to achieve United Nations Program on HIV and AIDS (UNAIDs) goals [25]. Near to one in two (47.7%), individuals changed their regimen in this present study. The regimen change rate found in the current study is higher than findings elsewhere in Ethiopia [12, 26]. It is also higher when compared to other cohort studies in Uganda (39.21%) [27] and Nigeria (28%) [28]. However, cohort studies from resource-rich countries like UK, Italy, and Brazil showed higher incidence rates of regimen changes (28.3–41.5 per 100 PY) [17, 29, 30]. This is expected as viral load measurement and availability of subsequent antiretroviral treatment options are limited when compared with resource-rich settings [31].

Toxicities were the predominant reasons for ART regimens changed. This is consistent with other studies [29, 30, 32–34]. Like other studies, [17, 27, 35] our study found that fat changes and peripheral neuropathy were the most frequent toxicities. Unlike our setting and other sub-Saharan countries, hematological toxicities were the most common toxicities reported in Latin Americans [16, 31, 36, 37].

New TB drug treatment was also a major reason for the initial ART regimen change, especially for NVP-based regimens. This was due to an interaction of NVP with the anti-TB drugs such as Rifampin [38, 39]. In order to avoid this drug interaction, clinicians replace the NVP with EFV [23, 24]. Being infected with HIV virus may also expose patients to develop TB early [38, 40] and thereby lead to modification of NVP to EFV.

In the current study, the hazard of regimen change was higher in Tb/HIV co-infected patients than in patients with HIV infections alone. This is consistent with other studies [20, 41, 42]. Studies showed that co-morbidities in patients with advanced disease and concurrent treatments for opportunistic diseases may affect antiretroviral tolerance and thereby disallow patients from regular treatment intake [40, 43, 44].

EFV-based regimens had the lower hazard of change than NVP-based regimens. This is similar to other studies [14, 16, 17, 45]. The increased hazard for NVP-based regimens change might be due to the frequent use of fixed-dose combinations containing D4T and NVP as D4T was the drug most often modified. Early modification of NVP due to new TB treatment was also a possible reason for an increased hazard of NVP-based regimen changes [38, 39].

Patients who were above the primary level of education had the higher hazard of regimen change than those below primary level education. Although we did not assess their knowledge, this might be due to better awareness of ART-associated toxicities and reasons for regimen change among the educated groups.

## Conclusion

One in two patients changed their ART, mainly due to drug toxicity. New TB drug treatment, being pregnant and treatment failure were other reasons for the regimen changed. Regimen changed patients were more likely to be HIV/TB co-infected, above primary level education, and on D4T or NVP based regimens. We recommended strengthening adherence level and adopting benchmarking programs such as a linkage-case-management to enhance ART linkage and retention, which have demonstrated to be effective in similar settings [46, 47].

## Limitation of the study

We acknowledge the limitations of the current study including:

- Reasons for regimen change were documented only if they were incidental to regimen change, therefore, their frequency cannot be used to estimate their actual occurrence.
- The outcome status of regimen changed patients was not known.
- Some variables such as mental illness and stigma were not assessed as our data source lacked this information.

## Abbreviations

ART: antiretroviral therapy
D4T: stavudine
EFV: efavirenz
FMOH: Ethiopian Federal Ministry of Health
HAART: highly active antiretroviral therapy
HIV/AIDS: human immunodeficiency virus/acquired immunodeficiency syndrome
IQR: interquartile range
JUTH: Jimma University Tertiary Hospital
LTFU: lost to follow up
NNRTIs: non-nucleoside reverse transcriptase inhibitors
NRTIs: nucleoside reverse transcriptase inhibitors
NVP: nevirapine
PCP: *Pneumocystis jiroveci* pneumonia
PMCT: prevention of mother to child transmission
TDF: tenofovir
UNAIDs: United Nations Program on HIV and AIDS
WHO: World Health Organization
ZDV: zidovudine
